# Field‐Driven Activation of Solid‐State Devices in Open Circuits for Energy Harvesting and Wireless Sensing

**DOI:** 10.1002/advs.75200

**Published:** 2026-04-07

**Authors:** Renyun Zhang, Magnus Hummelgård, Henrik Andersson, Nicklas Blomquist, Jonas Örtegren, Hans‐Erik Nilsson, Zhong Lin Wang

**Affiliations:** ^1^ Department of Engineering Mathematics and Science Education Mid Sweden University Sundsvall Sweden; ^2^ Beijing Institute of Nanoenergy and Nanosystems Chinese Academy of Sciences Beijing People's Republic of China; ^3^ Department of Electronic Design Mid Sweden University Sundsvall Sweden

**Keywords:** energy harvesting, open circuits, solid‐state devices, time‐varying electric fields, wireless sensing

## Abstract

Time‐varying electric fields induce displacement currents through capacitive coupling, resulting in current continuity even in the absence of conduction paths. While capacitive coupling is known as a parasitic effect, its role in directly activating solid‐state devices in open circuits remains underexplored. Here, we demonstrate that externally generated, time‐varying electric fields—produced by triboelectric excitation or moving charged objects—can directly activate linear and nonlinear components, such as diodes, rectifiers, and LEDs, without a galvanic connection. A lumped‐element capacitive‐coupling model captures the observed dependencies *V_ab_* ∝ω and *V_ab_* ∝1/*r*, validated experimentally on both linear and non‐linear components. The resulting field‐driven activation enables energy harvesting in open circuits, multi‐channel control, and wireless sensing of human motion and mechanical vibrations over meter‐scale distances. This quasi‐static capacitive coupling operates in a distinct regime compared to resonant wireless power transfer, because it is drive by low‐frequency, motion induced electric field changes that generate transient displacement currents in floating circuits. The findings here enable contact‐free activation of electronic components through discrete energy transfer rather than traditional continuous power delivery.

## Graphical abstract

Time‐varying electric fields can activate solid‐state devices such as LEDs and capacitors for energy harvesting and powering in open circuits. The response of solid‐state devices to time‐varying electric fields caused by vibrations can be utilised for smart sensing of human motions and object vibrations.

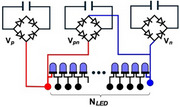

## 1 Introduction

Maxwell's incorporation of the displacement current amended Ampère's law, formalizing the principle of current continuity for regimes involving time‐varying electric fields that is independent of a conduction path [1, 2]. In the quasi‐static limit [3], this physics appears at the circuit level as capacitive coupling: time‐varying fields drive displacement currents through the surrounding dielectric [4]. Although capacitive coupling is well known as a parasitic or intentional effect in circuits [5], its role as a primary driver for activating functional solid‐state devices [6] in open‐circuit configurations remains underexplored. Recent work has demonstrated the ubiquity of electromagnetic fields from dynamic contact [7]. These works and other related approaches have been proven to work efficiently [8, 9]. However, the approaches rely on conduction currents in closed‐circuit configurations to deliver power to a load.

Recent advances in capacitive coupling have demonstrated efficient power transfer through the human body [6]. Their method relies on completing a galvanic conduction path through the body to deliver power. The study utilises the body as a wire in a closed system, while harnessing the body's inherent electric field as a remote control to drive floating components, enabling a new class of self‐powered, contactless sensing and control that operates entirely in an open‐circuit state. However, these approaches also ultimately rely on conduction currents in closed‐circuit configurations.

Triboelectric nanogenerators [10, 11] (TENGs) utilise capacitive coupling internally during charge separation and contact–separation cycles [12, 13], yet device operation ultimately relies on conduction currents in closed circuits. In contrast, we explore the direct use of quasi‐static capacitive coupling to drive floating two‐terminal devices—without any galvanic connection to the source of the field. In this framework, the time variation of the electric field induces displacement current [14, 15], creating current continuity even in the absence of physical charge transport across the gap. The response on the device is governed by the time derivative of the electric field (∂*E*/∂*t*), but not the electric field magnitude itself.

In this work, we experimentally demonstrate that time‐varying electric fields created by triboelectrification or a moving charged object can directly activate LEDs, rectifiers, and capacitors in open circuits. The observed signals scale inversely with distance (*r*) [16] and approximately linearly with the rate of field change ($\frac{dV_{S}}{dt}$) [17], consistent with the theoretical dependence, V $\propto \frac{1}{r}\frac{dV_{S}}{dt}$. We further extend the concept to demonstrate energy harvesting, position‐encoded control, contactless motion and vibration sensing. These results suggested a new operational mode for solid‐state electronics, which is field‐driven activation via quasi‐static capacitive coupling, equivalent to quasi‐static capacitive coupling continuity but observable in macroscopic devices under ambient conditions.

## 2 Results and Discussions

### 2.1 Activation of Solid‐State Devices

**FIGURE 1:**
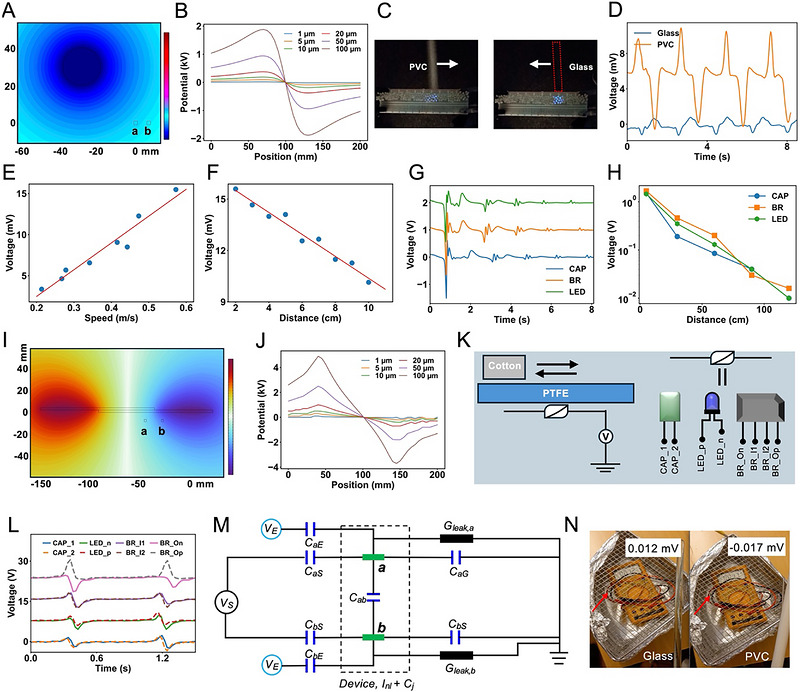
Activation of electronics by quasi‐static capacitive coupling. (A) A COMSOL simulation of the electric field of the charged circle at a charge density of ‐1 mC/m^2^, moving horizontally above the a and b that represent the two electrodes of the electronics. (B) Simulated potential difference between the a and b vs. the position of the circle. (C) Charged PVC and glass light up LEDs locally with opposite polarities. The area marked with a red dashed line indicates the position of the glass rod. (D) The voltage measured on an LED while a charged PVC and glass are moving horizontally above it, indicating opposite voltage. (E) A plot of the measured voltage vs. the moving speed of the PVC tube. (F) A plot of the measured voltage vs. the lateral distance of the PVC tube to the LED. (G) Measured voltages on a capacitor (CAP, 10 nF), a bridge rectifier (BR) and an LED while moving a PVC tube in front of them at different lateral distances. (H) A plot of the measured voltage vs. the lateral distance indicates that the intensity is inversely proportional to the distance. (I) A COMSOL simulation of the electric field while a piece of cotton slides on a piece of PTFE. (J) Simulated potential difference between the a and b vs. the position of the cotton. (K) A schematic drawing shows the voltage measurement of the electronics in open circuits, and the measured voltage signals are shown in (L). (M) A diagram shows the equivalent circuit of the activation of electronics with quasi‐static capacitive coupling. (N) Photographs show the response of a 10 nF capacitor inside a Faraday cage to the moving charged glass rod and PVC tube.

According to electric field principles, two points in an electric field could have different potentials [18]. If the electric field strength changes, the difference between the two points changes simultaneously, generating a quasi‐static capacitive coupling between the two points. Figure 1A shows a simulated image of a charge circle (to mimic a polyvinyl chloride, PVC tube used in the following experiments, charge density: ‐1 mC/m^2^) moving from left to right above two objects (SimV S1). The potential difference between the two objects facing each other was plotted in Figure 1B. The two surfaces (*a*, *b*) represent the two electrodes in diodes, LEDs, capacitors, etc. Results imply that a time‐varying electric field could generate quasi‐static capacitive coupling in electronics. Figure 1C illustrates capacitive activation of LEDs in open circuits by a moving charged PVC tube or a glass rod. When the negatively charged PVC or the positively charged glass rod moves near the device, the LED lights transiently, even though both terminals are floating. (Videos S1, see circuit in Figure S1). The observed polarity reverses with the sign of the moving charge, resulting in the LEDs lighting with opposite polarity. To prove it, we measured the voltage on an LED while moving a charged PVC in front of it (Figure 1D), and the result clearly shows the opposite response. Moreover, the induced voltage increases linearly (R^2^ = 0.95) with the moving speed of the tube (Figure 1E, see measured voltage signal in Figure S2) and decreases approximately *1/r* (R^2^ = 0.97) with lateral distance between the tube and the LED (Figure 1F, see measurement results in Figure S3), which is consistent with the dependence of capacitive coupling on field variation and separation. Results here demonstrated that the time‐varying field capacitively drives the floating device, activating it without any galvanic connection.

We have compared the responses of an LED, a capacitor (CAP) and a rectifier (RF) to a charged PVC that is moving in front of them at different distances (Figure 1G). Results have shown that the measured signal intensity of these three electronics was similar, and the intensity was inversely proportional to the lateral distance (*r*) of the PVC to the electronics (Figure 1H). This counterintuitive result can be explained by the dominance of the externally coupled capacitive flux in the floating configuration. The induced voltage is mainly by the geometry of the external electric field and the time variation of the source potential. Moreover, non‐linear devices such as LEDs and rectifiers show junction threshold effects and limited charge redistribution, which reduces the expected differences. More theoretical discussion is given below.

Besides the field generated by the charged object, the time‐varying electric field could also be generated by triboelectric excitation of materials in a similar way to operate triboelectric nanogenerators. Figure 1I shows a COMSOL simulation of the electric field created by rubbing a dielectric material (charge density: 100 µC/m^2^) on polytetrafluoroethylene (PTFE, charge density: ‐100 µC/m^2^) to mimic the rubbing of a piece of cotton on a PTFE tape that is attached to the backside of a breadboard (SimV S2). The potential difference between the two surfaces (a, b) is shown in Figure 1J. Experimentally, we have measured the potentials of the two terminals of a capacitor, an LED and a bridge rectifier in open circuits while activated by the triboelectric excitation (Figure 1K,L).

The quasi‐static capacitive coupling is generated by an electric field that is highly dependent on the size, geometry, and surface charge density of the moving object, like the PVC, which is a tube‐shaped object. Therefore, the activation effect is limited by the object, resulting in a localised effect. Video S2 shows an LED array lighting locally by one or two cotton strips working with the triboelectric activation mode. Simulation of the voltage across the LED array and the quasi‐static capacitive coupling is given in Figure S4, SimGif S1 and SimGif S2 in the supplementary information. According to the simulation, the LEDs should be lit only in one direction of the moving cotton strips because LEDs are a type of non‐linear electronics, and the direction of the quasi‐static capacitive coupling must match the junctions in the LEDs.

Figure 1M shows an equivalent circuit for the above cases. Here, we consider a floating two‐terminal device with node potentials [19], *V_a_* (*t*) and *V_b_* (*t*). A nearby source region (tribo interface or moving charged body) has potential *V_S_* (*t*). Each node forms capacitances to the source: *C_aS_*, *C_bS_*, to the ground: *C_aG_*, *C_bG_*, to the environment: *C_aE_*, *C_bE_*, and *C_ab_* between the nodes. The device branch current is *I_nl_* (*V_ab_*), with *V_ab_* = *V_a_*  − *V_b_*. In the quasi‐static limit (ω*L*/*c* ≪ 1), two equations can be obtained:

(1) $$\begin{array}{ccc} & & \left(C_{aS}+C_{aG}+C_{ab}+C_{aE}\right){\dot{V}}_{a}-C_{ab}{\dot{V}}_{b}+I_{nl}\left(V_{ab}\right)+G_{leak,a}\;V_{a}\\ & & =C_{aS}\;{\dot{V}}_{S}+C_{aE}{\dot{V}}_{E}+I_{noise,a}\left(t\right) \end{array}$$

(2) $$\begin{array}{ccc} & & \left(C_{bS}+C_{bG}+C_{ab}+C_{bE}\right){\dot{V}}_{b}-C_{ab}{\dot{V}}_{a}-I_{nl}\left(V_{ab}\right)+G_{leak,b}\;V_{b}\\ & & =C_{bS}\;{\dot{V}}_{S}+C_{bE}{\dot{V}}_{E}+I_{noise,b}\left(t\right)\; \end{array}$$

where *G*_*leak*,*a*_ and *G*_*leak*,*b*_ are the parasitic leakage conductance, *V_E_* is the environmental potential fluctuations, *I*_*noise*,*a*_(*t*) and *I*_*noise*,*b*_(*t*) are the stochastic noise current, ω  =  2π*f*(*f* is the frequency of the sliding), *L is* a characteristic size of the setup, and *c* is the speed of light. Equations (1) and (2) are the lumped‐element [20] manifestation of current continuity; the $C\;\dot{V}$ terms represent the displacement (capacitive) currents in the quasi‐static regime. To solve the differential voltage *V_ab_*, we linearise the device branch current [21] as *I*_nl_  ≈ *G_d_* *V_ab_*, apply a Fourier transform (replacing time derivatives with *j*ω, and solve the resulting system of linear equations for *V_a_* (ω) and *V_b_* (ω), which yields the frequency‐domain expression [22] for *V_ab_* (ω) = *V_a_* (ω) − *V_b_* (ω). The driven differential voltage becomes:

(3) $$V_{ab}\;\left(\omega \right)=\frac{j\omega \left[V_{S}\left(\omega \right)\left(C_{bG}C_{aS}-C_{aG}C_{bS}\right)+V_{E}\left(\omega \right)\left(C_{bG}C_{aE}-C_{aG}C_{bE}\right)\right]+\Delta I_{noise}\left(\omega \right)}{j\omega \left[\left(C_{aS}+C_{aG}+C_{aE}+C_{ab}\right)\left(C_{bS}+C_{bG}+C_{bE}+C_{ab}\right)-C_{ab}^{2}\right]+G_{eq}}\;$$

where *G_eq_* = *G_d_* (*C_aS_* + *C_aG_* + *C_aE_* + *C_bS_* + *C_bG_* + *C_bE_*) + *G_leak_*, Δ*I_noise_* (ω) = *I*_*noise*,*a*_ (ω) − *I*_*noise*,*b*_(ω). For a linear device ($G_{d}\to 0$), the small‐signal, frequency‐independent ratio simplifies to

(4) $$\begin{array}{c} V_{ab}\approx V_{S}\frac{C_{bG}C_{aS}-C_{aG}C_{bS}}{\left(C_{aS}+C_{aG}\right)\left(C_{bS}+C_{bG}\right)+C_{ab}\left(C_{aS}+C_{aG}+C_{bS}+C_{bG}\right)} \end{array}$$

For non‐linear devices [23], like LEDs and diodes, we incorporate the voltage‐dependent junction capacitance, *C_j_* (*V_ab_*):

(5) $$C_{j}\;\left(V_{ab}\right)=C_{j0}\;{\left(1+\frac{V_{ab}}{V_{bi}}\right)}^{-m}$$

where *C_j_* is the junction capacitance [24] and *V_bi_* is the built‐in potential. Therefore, we rewrite Equations (1) and (2) for non‐linear devices:

(6) $$\begin{array}{ccc} & & \left(C_{aS}+C_{aG}+C_{ab}+C_{aE}\right){\dot{V}}_{a}-C_{ab}{\dot{V}}_{b}+I_{nl}\left(V_{ab}\right)+C_{j}\left(V_{ab}\right){\dot{V}}_{ab}\\ & & +G_{leak,a}\;V_{a}=C_{aS}\;{\dot{V}}_{S}+C_{aE}{\dot{V}}_{E}+I_{noise,a}\left(t\right) \end{array}$$

(7) $$\begin{array}{ccc} & & \left(C_{bS}+C_{bG}+C_{ab}+C_{bE}\right){\dot{V}}_{b}-C_{ab}{\dot{V}}_{a}-I_{nl}\left(V_{ab}\right)-C_{j}\left(V_{ab}\right){\dot{V}}_{ab}\\ & & +G_{leak,b}\;V_{b}=C_{bS}\;{\dot{V}}_{S}+C_{bE}{\dot{V}}_{E}+I_{noise,b}\left(t\right) \end{array}$$

(8) $$\begin{array}{ccc} & & V_{ab}\approx V_{S}\\ & & \frac{C_{bG}C_{aS}-C_{aG}C_{bS}}{\left(C_{aS}+C_{aG}\right)\left(C_{bS}+C_{bG}\right)+\left(C_{ab}+C_{j}\right)\left((C_{aS}+C_{aG}+C_{bS}+C_{bG}\right))} \end{array}$$

The time‐domain behaviour in the equations reveals the fundamental scaling relationship. From Equation (3), we derive *V_ab_* ∝*j*ω*V_S_* (ω), leading to $V_{ab}\left(t\right)\propto \frac{dV_{S}}{dt}.$ Since source‐node capacitances scale as $C_{aS},\;C_{bS}\propto \frac{1}{r}$, the overall dependence becomes $|V_{ab}|\propto \frac{1}{r}\left|\frac{dV_{S}}{dt}\right|$. This theoretical prediction has been quantitatively verified by our experimental results above and below.

**FIGURE 2:**
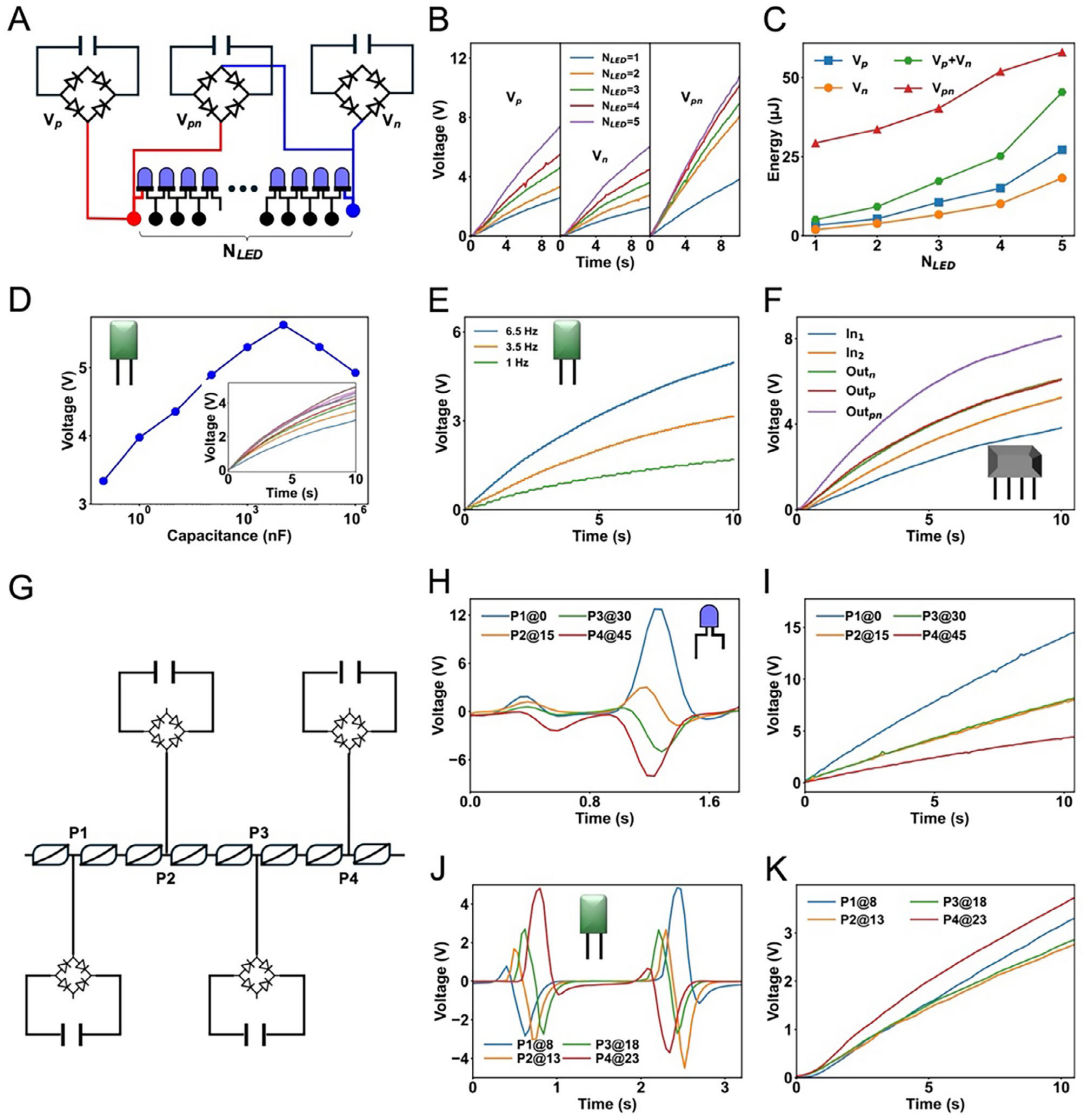
Energy harvesting on capacitively activated devices. (A) A schematic drawing shows the circuit for harvesting energy from serially connected LEDs. The operation mode is the same as in Figure 1k. In the case *V_p_*, a cable is connected from the *p* side of the LED array to the input of a rectifier. The other input of the rectifier is floating. In the case *V_n_*, the n side of the LED array was connected. In the case *V_pn_*, the p and n sides of the array were connected to the two inputs of the rectifier. The number of LEDs in the array was from 1 to 5. A 1 µF capacitor was connected to the rectifier to store the energy generated. (B) The voltage on the capacitor was measured for the three cases. (C) The energy output for the three cases during a 10‐s operation. The total energy of the case *V_p_* and *V_n_* was also plotted as (*V_p_ + V_n_*) to compare with the case *V_pn_*. (D) A plot of the voltage measured on the 1 µF capacitor after 10 s of operation for the experiments that replace the LED array with capacitors having capacitance ranging from 100 pF to 1 mF. Here, the two electrodes of the capacitors are connected to the two inputs of the rectifier. The insert in the Figure shows the charging curves. (E) Charge a 1 µF capacitor at different operation frequencies. Here, a 10 µF capacitor was used to be activated by the quasi‐static capacitive coupling in the same way as the LEDs. (F) A plot of the voltage measured on the 1 µF capacitor after 10 s of operation for the experiments that replace the LED with a bridge rectifier. *In_1_*, *In_2_*, *Out_p_* and *Out_n_* were operated in the same way as the case *V_p_* and *V_n_*. *Out_pn_* was operated in the same way as the case *V_pn_*. (G) A schematic drawing illustrates the energy harvesting at various connection points on electronic arrays of LEDs and capacitors. (H) The measured voltage at the positions in the circuit during one back‐and‐forth sliding circle. Here, an array of 62 serially connected LEDs was used. (I) Charge a 1 µF capacitor with the energy output from the four positions. (J) The measured voltage at the positions in the circuit during one back‐and‐forth sliding circle. Here, an array of 31 serially connected capacitors (100 nF) was used. (K) Charge a 1 µF capacitor with the energy output from the four positions.

The activation of electronic devices in this work is governed by transient energy transfer that is mediated by displacement current but not a continuous steady‐state powering. The motion of a charged source can induce current, $I_{d}=C_{eff}\;\frac{dV_{S}}{dt}$ that transfer charge $Q_{tr}=\int I_{d}dt$ to the floating capacitance of the electronics, where *C_eff_* is the effective capacitance between the source and the device. The corresponding transient device voltage can be estimated with *V_d_* ≈ *Q_tr_*/*C_ab_*, where *C_ab_* is the effective capacitance of the device. For LED that is a nonlinear component, it can be activated when the transient voltage exceeds its threshold (ca. 2∼3 V) that represented experimentally by the lightning of the LEDs. From energy point of view, Figure 2C has indicated that about 25 µJ electricity has been generated in 10 s for an array with 5 LEDs. An average power of 2.5 µW is sufficient for intermittent LED flashing, although it does not imply continuous bright operation of LEDs.

It should be noted that the mechanism here is different from that of capacitive wireless power transfer, electrostatic/capacitive sensors [25], Kelvin probe / non‐contact voltmeters [26], TENGs [27], and near‐field capacitive radio frequency coupling [28]. For instance, while recent studies have explored displacement current for wireless power [29] and non‐contact sensing [9], they function by inducing conduction currents in a closed receiver circuit. Our approach activates the floating device itself, with no closed circuit for conduction current. Table S1 in the supplementary information compares this work with other technologies. Another issue that needs to be addressed is how much the antenna effect [30] of the cables influences the signals. We tested the response of a capacitor (10 nF) directly connected to the terminal (Figure S5) of the data acquisition (DAQ) system and found that the signal is smaller than that of 50 cm cables between the capacitor and the DAQ (Figure S5). In our framework, this arises because longer conductors present a larger effective capacitance [31] to the source and to the environment, thereby increasing the external quasi‐static capacitive coupling coupled into the floating device. This is an electrostatic ‘antenna‐like’ effect in the quasi‐static regime, distinct from resonant radio frequency antennas [32] that couple to propagating waves.

To exclude the possibility that the signals is from electromagnetic radiation or direct conduction paths, we placed the battery‐powered sensing circuit (capacitor + multimeter) entirely inside a Faraday cage. Experimental results show that approaching a charged object outside still modulated the internal reading (Figure 1N). This behavior can be explained by a two‐stage electrostatic induction rather than direct field penetration. The charged PVC tube or glass rod induce a redistribution of charge on the cage surface, creating a time‐varying potential on the enclosure, which further generates an internal electric field that capacitively couples to the floating circuit. Due to unequal node‐to‐enclosure capacitances, the induced common‐mode signal is converted into a measurable voltage across the device. Therefore, the observed signal is from indirect capacitive coupling mediated by the enclosure, consistent with quasi‐static electrostatic principles. The interpretation is supported by results that no significant differences in the response were found where the cage was grounded or not. A video of the experiment is provided in Video S3.

### 2.2 Utilisation of the Activated Solid‐State Electronics

#### 2.2.1 Energy Harvesting in Open Circuits

Energy harvesting technologies typically require a closed circuit to guide the flow of electrons, which is common sense. However, we have demonstrated that energy can be harvested in open circuits, taking advantage of the activation of electronics by quasi‐static coupling. Figure 2A shows a schematic of three circuits used to study the energy output from LEDs (3 mm, blue light, serially connected) activated by quasi‐static capacitive coupling. In case *V_p_*, an input of a bridge rectifier is connected to the *p* side of LEDs, leaving the other input floating. The two outputs of the rectifier are connected to a capacitor (1 µF). In case *V_n_*, the n side of the LEDs is connected to the rectifier. In case *V_pn_*, the *p* and *n* sides of the LEDs are connected to the two inputs of the rectifier, which form a closed circuit for comparison. Figure 2B shows the measured voltage on the capacitor from the three cases. Results show that the case *V_p_* produces more energy than the case *V_n_*, which is, however, related to the number of LEDs in the open circuit. By comparing the energy output of the cases (Figure 2C), we found that the summary of the energy stored in the capacitor from cases *V_p_* and *V_n_* is smaller than that of case *V_pn_*. The difference between the outputs is due to the energy loss from parasitic capacitance leakage from the floating node to the environment that allows displacement current to partially leak into surrounding coupling paths; efficiency differences of the rectifier bridge under asymmetric driving particularly under low amplitude; the load effects of the measurement system itself including finite input impedance and capacitance. We have tried LEDs with different colours under the same conditions, and the results are similar.

In another experiment, we used capacitors instead of LEDs for energy harvesting in a similar manner. Results (Figure 2D) show that the energy output depends on the capacitance of the capacitors, with the capacitor having a capacitance of 10 µF generating the highest energy output, at a power of 1.6 µW. Moreover, the energy output was related to the frequency of the cotton sliding (Figure 2E). The harvested energy is related to the operation frequency and capacitance value, indicating a dependence on the derivative term *dV_S_*/*dt*. Although the absolute power is modest, the mechanism allows energy transfer without conduction paths or direct contact. For capacitors, no significant difference in energy output was found between the two terminals compared to the cases *V_p_* and *V_n_* for the LEDs, because capacitors are linear electronic components. Besides capacitors, we have also investigated the energy output from a bridge rectifier. Figure 2F shows the charging curve of a 1 µF capacitor by the rectifier with different connection ways. The circuit of the case (*In_1_*, *In_2_*, *Out_p_* and *Out_n_*) was connected in the same way as cases *V_p_* and *V_n_*, while the case *Out_pn_* was the same as the case *V_pn_*. Results show a similar phenomenon, where the case *Out_pn_* has the highest energy output compared to the case *V_pn_*, which is understandable because the bridge rectifier is composed of diodes.

In an array of LEDs or capacitors, the energy produced can be stored separately through parallel circuits. Figure 2G illustrates a circuit to store energy produced on the array in four capacitors (1 µF). In this construction, four connections were made at different positions in the array. In the case of using LEDs, 62 LEDs were serially connected on a breadboard, whereas 31 LEDs were used in the case of using capacitors. The electric potentials of the four connections vs. grounding during the operation process have been measured, and the results have indicated that the potential from the connections at the two sides (P1@0, P4@45 for LEDs, P1@8, P4@23 for capacitors) were higher than those in the middle (Figure 2H,J). The energy stored on the 1 µF capacitor after 10 s of operation has shown differences for LEDs (Figure 2I) and capacitors (Figure 2J), because LEDs are non‐linear electronic devices, whereas capacitors are linear electronic devices. For LED, the energy output from the two terminal connections (P1@0, P4@45) was significantly different, where the connection at P1@0 charged the capacitor to 14.5 V and the connection at P1@45 only charged it to 4.4 V, resulting in energy outputs of 105 µJ and 9.7 µJ, respectively. Summarising the energy output from the four connections, we obtained an energy density of 11.2 J/m^2^. For the capacitor array, the difference between the two terminal connections (P1@8 and P4@23) was not significant (Figure 2K) because of the linear nature of the capacitors. The sum energy density from the four connections was 2.5 J/m^2^.

#### 2.2.2 Open Circuit Powering and Position‐Encoded Control

**FIGURE 3:**
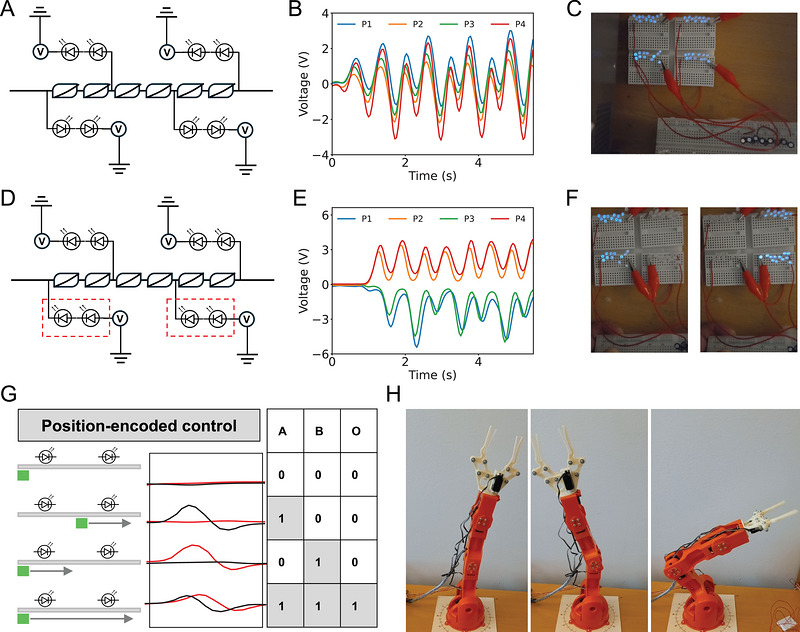
Open circuit powering and position‐encoded control. (A) A circuit shows the powering of four LED arrays by the energy generated on an open circuit. The open circuits were constituted of eight 10 µF capacitors. (B) The measured voltage at the four sub‐circuits. The operation mode is the same as described in Figure 1k. (C) A photograph showing the lighting of LED arrays powered by an open‐circuit capacitor array. (D) A circuit shows the powering of four LED arrays by the energy generated on an open circuit. Here, the LEDs in the red dash rectangle were placed opposite to those in (A). (E) The measured voltage at the four sub‐circuits. (F) A photograph showing the lighting of LED arrays powered by the open‐circuit capacitor array, illustrating the sensitivity of the lighting to the movement of the cotton sliding on the PTFE. (G) A position‐encoded control constructed by two LEDs. The LEDs were placed at two different places on a breadboard. A piece of cotton (the green square) was sliding on the PTFE film attached to the back of the breadboard. The starting point of the cotton's movement generated different signals that could be interpreted as a command. (H) Photographs of a robot arm controlled by the position‐encoded control, showing the three states after receiving the commands.

The above results demonstrate that we can harvest energy in an open‐circuit construction, which can be used to power other electronics. Due to its unique energy generation mode, we can power electronics in a way that differs from traditional ones. Figure 3A show a circuit for powering four LED arrays in parallel with energy generated on the eight‐capacitor array. One terminal of the LED arrays was connected at different positions (two at the terminals and two connections at the third and fifth capacitors) in the capacitor array, and the other terminals are grounded. The LED arrays were connected in the same direction, resulting in similar electric signals measured on the voltmeter (Figure 3B). Figure 3C shows a photograph of the four LED arrays lit up by the eight‐capacitor array in an open circuit. Through different connections (Figure 3D), we can control the ON/OFF state of the LED arrays in various ways. Figure 3E shows the potentials at the connection vs. the grounding, indicating the opposite signals of the two LED array connections. Due to the different connections, the LED arrays light differently while the cotton slides in different directions on the PTFE (Figure 3F). The results demonstrate that we can simultaneously power and control multiple devices using this approach.

Taking advantage of the LED's non‐linear nature, we have constructed a position‐encoded control for controlling a robot arm. Such a construction includes two LEDs inserted on a breadboard and PTFE tape on the back of the breadboard, as illustrated in Figure 3G. Here, by sliding a piece of cotton or just a finger (Video S4) on the PTFE at different starting points, we can create different electrical signals. Such electric signals were easily converted to commands to operate a robot arm (Figure 3H). Such a construction could be easily scaled up to control multiple devices with a single motion.

#### 2.2.3 Wireless Sensing of Human Activities for Security and Healthcare Applications

**FIGURE 4:**
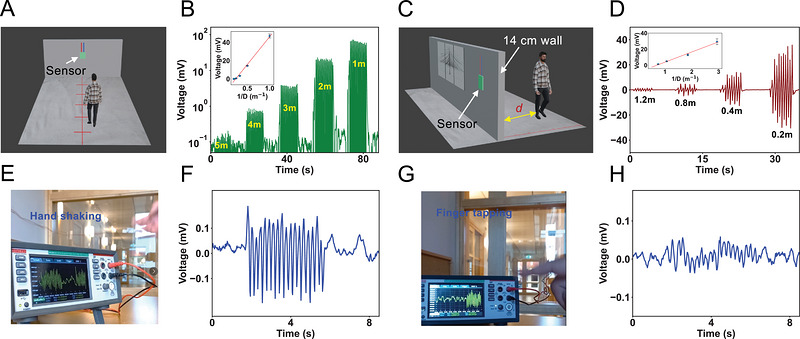
Wireless sensing of human activities. (A) An illustration of the sensing of stepping at different distances to the sensor attached to a wall (1.4 m from the floor). Illustration was made using Blender 3D and plugins. (B) Measured electric signals at different distances. The insert shows a plot of signal intensity vs. the inverse of distance (1/D). (C) An illustration of the sensing of stepping over a 14 cm wall. The sensor (a 10 nF capacitor) was attached to one side of a wall (1.4 m from the floor), and the human was stepping on the other side of the wall at different distances from the wall. Illustration was made using Blender 3D and plugins. (D) Measured the electric signals at different distances of the person from the wall. The insert shows a plot of signal intensity vs. the inverse of distance (1/D). (E) A photograph shows the sensing of hand shaking (to mimic the hand shaking of a person with Parkinson's disease) at a distance of 1 m from the sensor. (F) The measured electric signal on the sensor while hand shaking. (G) A photograph shows the sensing of finger tapping in the air at a distance of 1 m from the sensor. (H) The measured electric signal on the sensor while tapping a finger in the air.

We have demonstrated above that a charged tube can activate solid‐state electronics, where the tube is charged to a relatively high charge density. However, to activate solid‐state electronics, there is no need for a specifically charged object. In our previous studies, we have found that the human body generates triboelectric charge through motions [33, 34, 35], which creates an electric field surrounding the human body. Such an electric field was found to activate LEDs and capacitors, where electric signals were measured while a person performed motions near the electronics. In our experiment, we found that a 10 nF capacitor generates the best electric signal when activated. The background signal of the sensor placed on a wall was found at a range of ‐20 to 20 µV (Figure S6) without the presence of experimental participant in the room. To sense the activity, the participant stepping at 1, 2, 3, 4, and 5 m to the sensor. Results have shown that the sensor could detect a person's step from a distance of 5 m (Figure 4A; Figure S7), and the signal intensity (Figure 4B) was inversely proportional (R^2^ = 0.989) to the distance between the human body and the capacitor, as shown in the inset in Figure 4B. The signal‐to‐noise ratio (SNR) were found to be 60.2, 49.9, 36.8, 22.3, and 11.4 dB (Figure S8), indicating that the SNR is sufficient for threshold‐based detection. Such an indoor activity sensing strategy is self‐powered and low‐cost. It even worked when the person and the sensor were separated by a 14 cm wall (Figure 4C; Video S5). Figure 4D shows the measured voltage on the sensor at various distances between the human and the wall, and the insert indicates a linear relationship (R^2^ = 0.99) between the intensity and the inverse of the distance (1/D). In these two cases, the triboelectric charge change on the body was complex because it was influenced by the charges generated between the shoe and the floor, the insole and the socks, and so on. However, it is the overall charge on the human body that plays the role in activating the sensor. Nevertheless, the findings indicated that this sensing strategy can detect human motion at a distance. Such results indicate the potential of the strategy for indoor security applications, as it operates at a distance and effectively across a wall, and the sensor is self‐powered, requiring no external power sources.

In another study, the person stood still 1.0 m from the sensor while shaking his hand to mimic Parkinson's disease (Figure 4E). The electric signal measured on the sensor showed that hand shaking could be detected wirelessly (Figure 4F). The sensor could also sense single‐ finger motions at the same distance (Figure 4G,H). Both the intensity and the frequency of the hand shaking and finger tapping were sensed, which are important parameters for evaluating neural diseases [36, 37]. Surprisingly, it could sense the the movement in shoes at 1.0 m away (Video S6). Such a sensitivity could be applied for real‐time monitoring of physical function‐related diseases such as Parkinson's disease [36]. Compared to other methods [38, 39, 40], our approach is based on a self‐powered sensor that operates wirelessly, requires no wearable devices, and is low‐cost, which significantly enhances comfort and accessibility.

#### 2.2.4 Wireless Sensing of Objects and Mechanical Vibrations

**FIGURE 5:**
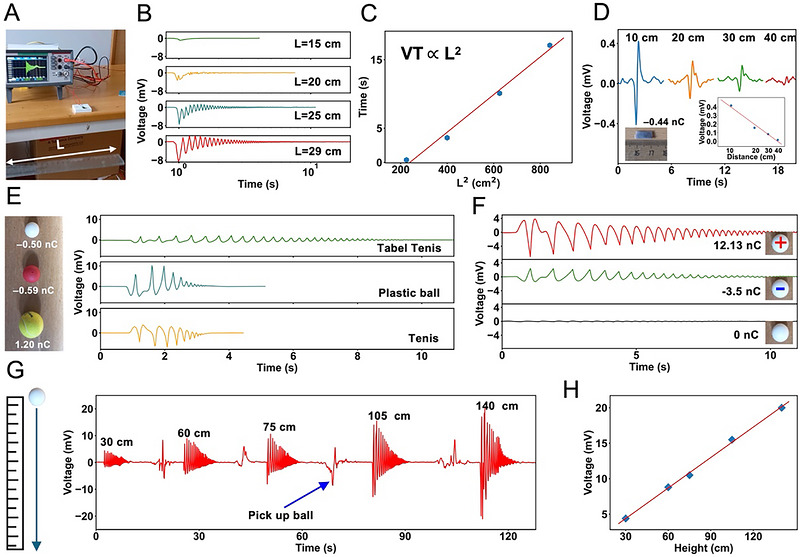
Wireless sensing of objects and mechanical vibrations. (A) A photograph of measuring the vibration of a ruler. (B) The measured electric signals of the ruler at different *L* values. The displacement of the free terminal was 8 cm for all experiments. (C) A plot of the vibration time vs. *L^2^*. (D) The electric signals were measured while dropping a piece of PTFE (15 mm × 5 cm, surface charge: ‐0.44 nC) at lateral distances to a capacitively activated sensor (a 10 nF capacitor) of 10, 20, 30, and 40 cm. The height of the starting position was 1.4 m. The insert shows a plot of the voltage vs. the lateral distances. (E) Identify different balls (table tennis, plastic ball, and tennis ball) with the sensor, showing a photograph of the balls, the total surface charge of the balls (‐0.50 nC for the table tennis ball, ‐0.59 nC for the red plastic ball, and 1.20 nC for the tennis ball) and the measured electric signals after dropping the ball at 1.4 m from the floor. (F) Determine the charge states of a table tennis ball. The table tennis was charged by rubbing it with PTFE, cotton and wet tissue, which gave the total surface charge of 12.13 nC, ‐3.5 nC and 0 nC, respectively. The lateral distance of the balls to the sensor was 20 cm. (G) Sensing the starting height of the table tennis ball, showing the measured electric signals. The signals in the Figure that are similar to the one pointed by the blue arrow are generated by picking up the ball from the floor. (H) A plot of the measured voltage vs. the starting height of the table tennis ball. The total charge of the ball was ‐0.65 nC, and the lateral distance to the sensor was 10 cm.

In our study, we have found that a capacitor could be activated by the movement of objects. In the first experiment, we placed a ruler in front of a 10 nF capacitor as a sensor (lateral distance: 10 cm) and allowed the ruler to vibrate at different free vibrating lengths (*L*). Figure 5A shows a photograph of the experimental setup, where one side of the ruler was fixed. Figure 5B also illustrates the measured electric signals from the sensor (a video capturing the experiment is provided in Video S7), indicating the sensor's sensitivity to vibration. According to physical principle [41, 42], the total vibration time should be proportional to the square of *L*, and it has been proved by our experimental results (R^2^ = 0.97, Figure 5C).

Figure 5D shows the measured electric signal by dropping a 15 mm × 5 mm‐sized PTFE piece (The total charge on the PTFE was ‐0.44 nC) after being charged with cotton (Video S8). The sensor is positioned 70 cm above the floor, and the PTFE was dropped from a height of approximately 1 m, 10, 20, 30, and 40 cm away (lateral distance) from the sensor. The insert in Figure 5D shows that the measured signal intensity was inversely proportional (R^2^ = 0.97) to the lateral distance. In this case, the PTFE is not bouncing after being dropped on the floor; therefore, only one negative and one positive peak were observed. If different balls were used, the bouncing of the ball on the floor could be detected in real‐time (Figure 5E). Each time the ball bounced on the floor, a charge transfer occurred that changes the surface triboelectric potential. Different balls have different surface charges and mechanical properties that decide the numbers of bouncing, which lead to distinct bouncing behaviors that can be easily identified. We used a table tennis ball (‐0.50 nC), a plastic ball (‐0.59 nC), and a tennis ball (1.2 nC) in the experiment, and the electric signals measured were significantly different. Such a detection even worked when balls were at different charge states. Here, we charged a table tennis ball to different states (12.13, ‐3.5 and 0 nC). Results have clearly shown that the electric signals were in opposite directions if the ball were charged oppositely (Figure 5F). The detection also worked for a ball dropping at different starting heights (Figure 5G,H). A linear relationship (R^2^ = 0.998) has been obtained between the signal intensity and the heights, which correlates with the potential energy of the ball. The above results indicated that, based on the electric signal generated by the capacitively activated electronics, different objects and their states can produce distinguishable signals.

## 3 Conclusions

In summary, we report a finding that solid‐state electronics, such as LEDs, capacitors, and rectifiers, can be activated by quasi‐static capacitive coupling in open circuits. Taking advantage of the activation, energy harvesting could be achieved. Moreover, we have demonstrated the utilisation of the activation for powering electronics, position‐encoded control, and different types of sensing, including human body motion and mechanical vibrations. The findings presented here demonstrate the effectiveness of quasi‐capacitive coupling‐driven electronics activation and its broad applications, ranging from energy harvesting and powering to security control, healthcare, mechanical vibration monitoring, and object identification. However, the system can be susceptible to environmental electromagnetic interference, necessitating careful signal conditioning in noise environments. Future studies will be directed toward optimising the field source, investigating phenomena at high frequencies, and developing integrated device designs to mitigate the abovementioned limitations. For use as sensors, artificial intelligence (AI) technologies are highly needed for analysing data in practice, as real application scenarios are more complex and manual feature extraction becomes very difficult.

## 4 Methods

All diodes, LEDs and capacitors were purchased from Elfa. The PTFE was purchased from High Tech Flon (Germany).

### 4.1 Simulation

All simulations were performed using COMSOL Multiphysics version 6.2. To simulate the voltage difference across the nodes, two metal wires with 0.01 mm in between were placed in the simulation model.

### 4.2 Electric Measurements

Electric measurements were performed using a Keithley 6510 DAQ system with a sampling rate of 50 /s (NPLC = 1). Such a ratio is selected because it cancels out the effect of the AC noise. For multichannel measurement, a 7700 multiplexer module was connected to the DAQ. For the electric measurements in Figures S2, S3, and S5, a PXI 4071 digital multimeter (National Instruments) with a sampling rate of 200 000 samples per second was used. The total surface charges of objects were measured using a system consisting of a Faraday cup and a Keithley 6514 electrometer.

For the experiments in Figure 1, the LEDs, diodes, and capacitors were placed on a breadboard, and one of the devices' legs was connected to the Keithley 6510 DAQ. A piece of fur and a piece of silk textile charged the PVC tube and the glass rod. For the experiments presented in Figure 1k and thereafter, a PTFE tape was attached to the backside of the breadboard (after removing the original attached tape on the breadboard), and a piece of cotton (purchased from VWR) was used to rub the PTFE to generate the time‐varying electric field. The room temperature was about 20°C, and the humidity level was about 50∼60% when performing these experiments.

For the measurement of body motions, hand shaking, and finger tapping. The room temperature was approximately 20°C, and the relative humidity level was around 50%. No other persons were present during the experiments.

## Funding

This project is financially supported by the Swedish Research Council, the Knowledge Foundation of Sweden.

## Conflicts of Interest

RY Zhang has submitted a US patent application (application number: 19/362,475) and a Chinese patent application (Application number: 202511491096.0) based on the finding here.

## Supporting information




**Supporting File 1**: advs75200‐sup‐0001‐SuppMat.docx.

**Supporting File 2**: advs75200‐sup‐0002‐VideoS1.mov.

**Supporting File 3**: advs75200‐sup‐0002‐VideoS2.mov.

**Supporting File 4**: advs75200‐sup‐0004‐VideoS3.mov.

**Supporting File 5**: advs75200‐sup‐0005‐VideoS4.mov.

**Supporting File 6**: advs75200‐sup‐0006‐VideoS5.mov.

**Supporting File 7**: advs75200‐sup‐0007‐VideoS6.mov.

**Supporting File 8**: advs75200‐sup‐0008‐VideoS7.mov.

**Supporting File 9**: advs75200‐sup‐0009‐VideoS8.mov.

**Supporting File 10**: advs75200‐sup‐0010‐SimGifS1.mov.

**Supporting File 11**: advs75200‐sup‐0011‐SimVS1.A.mov.

**Supporting File 12**: advs75200‐sup‐0012‐SimGifS2.A.mov.

**Supporting File 13**: advs75200‐sup‐0013‐SimVS2.A.mov.

## Data Availability

The data that support the findings of this study are available from the corresponding author upon reasonable request.
