# Supplementary material for: Field‐Driven Activation of Solid‐State Devices in Open Circuits for Energy Harvesting and Wireless Sensing
Source: Adv Sci (Weinh). 2026 Apr 7:e75200. Online ahead of print. doi: 10.1002/advs.75200 (PMC13334643; doi:10.1002/advs.75200)
Supplement: Supplementary file 1 — Supporting File 1: advs75200‐sup‐0001‐SuppMat.docx. [file ADVS-9999-e75200-s004.docx]

**Supporting Information**

**Field-driven activation of solid-state devices in open circuits for energy harvesting and wireless sensing**

*Renyun Zhang*, Magnus Hummelgård, Henrik Andersson, Nicklas Blomquist, Jonas Örtegren, Hans-Erik Nilsson, Zhong Lin Wang**

Figure S1. The circuit of lighting up LEDs with a charged PVC tube or a glass rod. The LEDs are inserted on a breadboard according to the circuit, and no connections are built between the two terminals, resulting in an open circuit.

Figure S2. Measured voltage signal when moving a charged PVC tube back and forth in front of an LED at different average speeds.

Figure S3. Measured voltage signal when moving a charged PVC tube back and forth at different lateral distance from the LED.

Figure S4. Simulated LED illumination activated by sliding one or two cotton strips on PTFE. (a) LED illumination occurs when the motion-induced voltage polarity forward-biases the LED junction. (b) No illumination occurs when the induced voltage polarity reverse-biases the junction. (c) Simultaneous motion of two strips illuminates the LEDs when the combined voltage forward-biases the junction. (d) No illumination occurs when the combined voltage from two strips reverse-biases the junction.

Figure S5. The voltage signal that was measured on a 10 nF capacitor that is directly connected to a DAQ (bottom) and via a 50 cm cable (top).

Figure S6. Background noise signal of the sensor attached to a wall in a room without the presence of any person.

Figure S7. Signal generated from stepping at 5 m away from the sensor versus the background signal while the person stands still.

Figure S8. A plot of the SNR versus the distance (D) between the experimental participant and the sensor.

Table S1. Comparison of the method in this work with other technologies.

| Method | Coupling path | Needs galvanic return? | Typical range (m) | Notes / Representative references (DOI) |
| --- | --- | --- | --- | --- |
| This work | Field-driven displacement (capacitive) | No | ~5 | Localized, low-power control without conduction path — demonstrated herein. |
| Capacitive WPT | Capacitive plates / dielectric field coupling | Often (required return plate) | 0.05 – 0.3 | High-power intentional coupling; limited by parasitics and misalignment. |
| NFC / RF | Magnetic / electric near-field resonance | No (RF) | ≤ 0.05 (near field) – 0.2 (coil pair) | Tuned inductive or resonant coils; standard for identification and payment tags. |
| TENG | Contact-separation triboelectric / conduction | Yes (to load) | < 0.01 (contact) | Converts contact-induced charge transfer into conduction current; requires closed load circuit. |
| Kelvin probe / non-contact voltmeter | Electrostatic coupling (capacitive modulation) | No | 10⁻³ – 10⁻² | Measures surface potential by detecting modulated capacitance; metrological, not energy-transfer capable. |
